# Line-Scanning Particle Image Velocimetry: An Optical Approach for Quantifying a Wide Range of Blood Flow Speeds in Live Animals

**DOI:** 10.1371/journal.pone.0038590

**Published:** 2012-06-26

**Authors:** Tyson N. Kim, Patrick W. Goodwill, Yeni Chen, Steven M. Conolly, Chris B. Schaffer, Dorian Liepmann, Rong A. Wang

**Affiliations:** 1 Laboratory for Accelerated Vascular Research, Division of Vascular Surgery, Department of Surgery, University of California San Francisco, San Francisco, California, United States of America; 2 Department of Bioengineering, University of California, Berkeley, California, United States of America; 3 Department of Biomedical Engineering, Cornell University, Ithaca, New York, United States of America; University of Arizona, United States of America

## Abstract

**Background:**

The ability to measure blood velocities is critical for studying vascular development, physiology, and pathology. A key challenge is to quantify a wide range of blood velocities in vessels deep within living specimens with concurrent diffraction-limited resolution imaging of vascular cells. Two-photon laser scanning microscopy (TPLSM) has shown tremendous promise in analyzing blood velocities hundreds of micrometers deep in animals with cellular resolution. However, current analysis of TPLSM-based data is limited to the lower range of blood velocities and is not adequate to study faster velocities in many normal or disease conditions.

**Methodology/Principal Findings:**

We developed line-scanning particle image velocimetry (LS-PIV), which used TPLSM data to quantify peak blood velocities up to 84 mm/s in live mice harboring brain arteriovenous malformation, a disease characterized by high flow. With this method, we were able to accurately detect the elevated blood velocities and exaggerated pulsatility along the abnormal vascular network in these animals. LS-PIV robustly analyzed noisy data from vessels as deep as 850 µm below the brain surface. In addition to analyzing *in vivo* data, we validated the accuracy of LS-PIV up to 800 mm/s using simulations with known velocity and noise parameters.

**Conclusions/Significance:**

To our knowledge, these blood velocity measurements are the fastest recorded with TPLSM. Partnered with transgenic mice carrying cell-specific fluorescent reporters, LS-PIV will also enable the direct *in vivo* correlation of cellular, biochemical, and hemodynamic parameters in high flow vascular development and diseases such as atherogenesis, arteriogenesis, and vascular anomalies.

## Introduction

A functional vascular supply is critical for efficient transport of nutrients, fluids, signaling molecules, and circulating cells between tissues and organs in the vertebrate system. Lack of appropriate vascular development and maintenance contributes to the pathogenesis of many diseases and can involve excess angiogenesis, abnormal remodeling, and insufficient growth or regression of vessels [1], [2], [3]. Importantly, hemodynamic forces have long been recognized to play a defining role in shaping blood vessels [4], [5] and in mediating vascular disease. Surgical manipulation of blood flow can cause abnormalities in heart and vessel development [6], [7] and primary defects in heart contractility have detrimental effects on vessel and cardiac remodeling [8], [9], [10]. Recent studies have also identified genetic programs involved in specifying arteries and veins prior to circulation [11], [12], raising important questions on the interplay of genetic and hemodynamic cues in arteriovenous development and plasticity. This interplay is enigmatic yet critical toward understanding vascular diseases such as arteriovenous malformation (AVM), an often dangerous condition characterized by enlarged, tangled shunts that bypass normal capillary beds. These high-flow lesions are believed to persist and enlarge under the influence of hemodynamic forces [13], [14], yet little direct evidence links flow with cellular and molecular mechanisms for their pathogenesis. The ability to correlate blood flow with cellular and molecular-level dynamics in living animals would improve our understanding of vascular development and disease. However, a key obstacle has been the inability to quantify high blood velocities in individual vessels deep in living specimens with concurrent high-resolution imaging of vascular cells.

Several well-validated methods have been used to quantify flow within microvessels [15], [16], [17] and microfluidic devices [18], [19] based upon cross-correlation analyses of particle motion. The two-slit photometric method utilizes the irregular spacing of red blood cells (RBCs) moving single-file through a capillary vessel. The pattern produced by these cells as they cross a stationary point remains relatively constant over short distances, and can be used to determine the transit time of RBCs between two detection points separated by known distance along a vessel [15], [16]. More recently, video-rate approaches have been used to determine the displacement of particles between sequential two-dimensional images given sufficient acquisition rates [18], [19]. Importantly, *in vivo* implementations of these approaches have generally been limited to vessels that can be isolated in thin or surface preparations due to technical challenges in acquiring image data with sufficient spatiotemporal resolution deep in tissues. For this reason, two-photon laser scanning microscopy (TPLSM) has been the tool of choice for studying hemodynamics at high resolution in mice and rats [20], [21], because it can penetrate hundreds of micrometers by mitigating the effects of light-scattering on image generation [22], [23], [24]. However, this depth imaging ability comes at the cost of imaging speed and has hindered the quantification of faster blood velocities. Prior investigations using TPLSM have generally focused on blood velocities under 10 mm/s, although one study reported analysis up to 35 mm/s [25]. The adult mouse harbors far greater blood velocities throughout the body that can exceed 180 mm/s in the carotid artery [26] and 500 mm/s in the suprarenal aorta [27], [28]. The ability to analyze a large range of blood velocities in mice with diffraction-limited resolution and deep imaging would enable detailed correlation of cellular, molecular, and local hemodynamic cues in high-flow processes such as atherogenesis, arteriogenesis, and AVM.

TPLSM and confocal line-scan geometries have been used to track RBC motion in a vessel-specific [20], [29], [30], [31], [32] or multi-vessel [33] fashion. Line-scanning can acquire substantially more frames per second than two-dimensional imaging and, when oriented along the length of a vessel, can resolve RBC motion over relatively large distances. Typically, sequential scans are visualized as a space-time image with distance on the abscissa and time advancing downward on the ordinate. The blood plasma is loaded with fluorescent dye, allowing individual RBCs to appear as dark spots. These dark spots produce streaks in the space-time data if there is sufficient overlap of the same RBCs between scans [20], [34]. The slope of each streak is inversely proportional to the cell’s speed and previous methods have quantified moderate blood velocities by determining the angle of the streaks [20], [29]. Alternatively, velocity can be calculated from the Euclidean distance traversed by the centroids of individual RBCs in consecutive line-scans, divided by the time between scans [35]. However, as the velocities of the RBCs increase relative to the line-scanning rate, individual cell are difficult to track and no longer generate discernable streaks that can be analyzed be these approaches.

To overcome this limitation, we developed a method to analyze a significantly wider range of velocities using TPLSM or confocal microscopy. Line-scanning particle image velocimetry (LS-PIV) determines the RBC displacements between pairs of line-scans using spatial cross-correlation analysis. This approach enables quantification of blood velocities from capillaries to high-flow abnormalities deep within the living animal. Here we describe the operating principles of LS-PIV and test its robustness to noise and increasing blood flow speed. We demonstrate the utility of this technique by quantifying blood flow velocities with high temporal and spatial resolution within a mouse model of brain AVM [36], [37].

**Figure 1:**
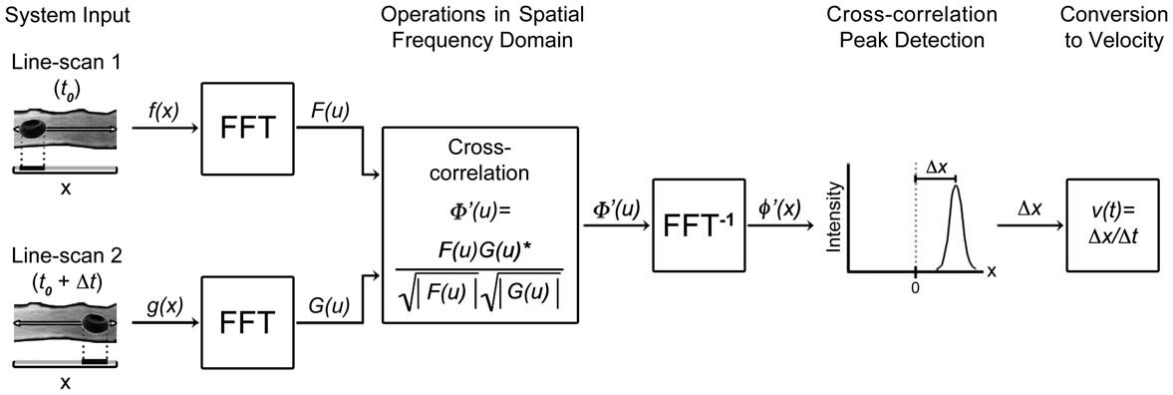
Illustration of generalized LS-PIV. RBCs appear dark amid fluorescently labeled plasma. Two successive line-scans are recorded along the central axis of the vessel, capturing the displacement of an RBC across time interval Δ*t.* The line-scans are Fourier-transformed, cross-correlated with a symmetric phase-weighted filtering operation, and inverse Fourier-transformed. A peak detection operation is performed to locate the correlation peak corresponding to the RBC displacement, which is converted to velocity by dividing by the time interval between the scans.

**Table 1:** LS-PIV Algorithm.

Step	Description
1	Record line-scan data along the axis of vessel.
2	Convert data to a space-time image (see Figure S2) to form image s_1._
3	Subtract the time-averaged background.
4	Section out the region of interest in the space-time data to form image s_2._
5	1D Fourier transform the space-time images s_1_ and s_2_ along the spatial axis to form the Fourier domain space-time matrices S_1_ and S_2_, respectively.
6	Resample the Fourier domain space-time matrix into reference-scan and ( 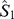 ) line-scan ( 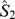 ) images. 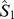 and 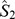 are two representations of the same space-time matrix, but with 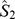 delayed by one or more time steps. Fast flows typically require a delay of only one time step, while slower flows may require a delay of multiple time steps.
7	Construct the symmetric phase-weighted filter to reduce cross-correlation artifacts [44].
	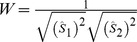
8	Perform cross-correlation and phase only filter in Fourier space.
	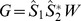
9	Inverse Fourier transform the cross-correlated, phase-filtered signal G along the spatial frequency axis to result in the spatial domain LS-PIV image.
10	Fit Gaussian peaks to the filtered LS-PIV to solve for spatial shift with sub-pixel resolution.
11	Convert the detected pixel shift into RBC velocity.

## Methods

Additional experimental details are provided in Text S1. Example data-sets and Matlab code are available at: https://sourceforge.net/projects/lspivsupplement/files/.

### Mice

This study was carried out in strict accordance with NIH regulations and the Institutional Animal Care and Use Committee at the University of California San Francisco. The protocol was approved by the IACUC at UCSF (Approval Number: AN085404-01). *Tie2-tTA* and TRE-Notch4* mice were generated by our lab as previously described [36] and crossed to produce mice harboring brain AVMs [37]. Tetracycline sucrose water (0.5 mg/mL Tetracycline hydrochloride, Sigma, and 50 mg/mL D-sucrose, Fisher BioReagents) was administered to pregnant mothers and withdrawn when pups were born to allow expression of endothelial Notch4* in neonates. Wild-type mice were used to study normal brain vasculature. *Ephrin-B2^+/H2B-eGFP^* mice [38] were kindly provided by the Soriano lab and aided with *in vivo* visualization of arterial endothelium with cellular resolution. Mice were backcrossed into FVB/N by nine generations or greater.

### In Vivo Imaging

TPLSM imaging of mouse brain was performed as previously described [39]. In brief, three week-old mice were anesthetized with 0.5–2% isoflurane in 2 L/min of oxygen and warmed with a homeothermic blanket (Harvard Apparatus). The head was immobilized with a stereotax (myNeuroLab.com**)** for surgery and imaging. The scalp was treated with betadine and given subcutaneous injection of 0.125% bupivacaine prior to surgery. A small craniotomy was performed over the right cortex, bathed in artificial cerebral spinal fluid, covered with a 5 mm coverglass (World Precision Instruments), and sealed with dental acrylic (Lang Dental) to provide optical access to the brain. Mice were administered with subcutaneous injections of 0.1 mg/kg buprenorphine pre-operatively and twice daily for three days. For imaging, the blood was fluorescently labeled with 2000 KDa Texas Red-dextran that was prepared according to a published protocol [40] and filtered with 1000 KDa dialysis tubing (Spectrum Labs).

Images and line-scan data were obtained with a locally constructed two-photon laser scanning microscope using a long working distance 1.0 NA water dipping objective (Zeiss) and MPScope 1.0 software [41] to control the microscope and record data (Figure S1). Texas Red and GFP fluorescence were excited simultaneously using low-energy 100 femtosecond laser pulses at 80 MHz, centered at 870 nm, generated with a Titanium:Sapphire laser oscillator (Mai Tai HP, Newport Spectra-Physics) that was pumped by a continuous wave diode laser (Millenia; Newport Spectra-Physics) and passed through a dispersion compensator (DeepSee; Newport Spectra-Physics). Intensity was controlled by rotating a λ/2 wave-plate relative to a polarizer and utilizing the transmitted beam. The laser was then routed onto a microscope platform based on a previous design [42] that was optimized for deep tissue imaging and blood velocity measurements. The laser pulses were raster-scanned by galvanometric mirrors (Cambridge Technology) and relay-imaged to the back aperture of the objective. Fluorescence was collected by the same objective, reflected by a primary dichroic mirror (700 nm long-pass; Chroma), split into red and green channels with a secondary dichroic mirror (560 nm long-pass; Chroma), spectrally cleaned with additional band-pass filters (Chroma), and relayed to photomultiplier tubes (PMT; H7422P-40MOD; Hamamatsu). The PMT signal was amplified (10^5^ transimpendence gain), low-pass filtered (4 pole Bessel at f_c_  = 300 kHz), and digitized. The anesthetized mouse’s head was immobilized with a stereotax and placed on a three-dimensional translation stage (11SI70521 Rev. 01; Newport) that allowed precise positioning of the brain relative to the imaging focus. Two-photon excited fluorescence was generated within the focal volume, enabling diffraction-limited resolution deep in tissue [24]. On our system, this corresponds to ∼0.4 µm in the image (‘xy’) plane and ∼1.5 µm along the optical (‘z’) axis by calculation [24]. Image or line-scan data was generated by rapidly scanning the focal volume throughout the image field or along a defined path, respectively.

**Figure 2:**
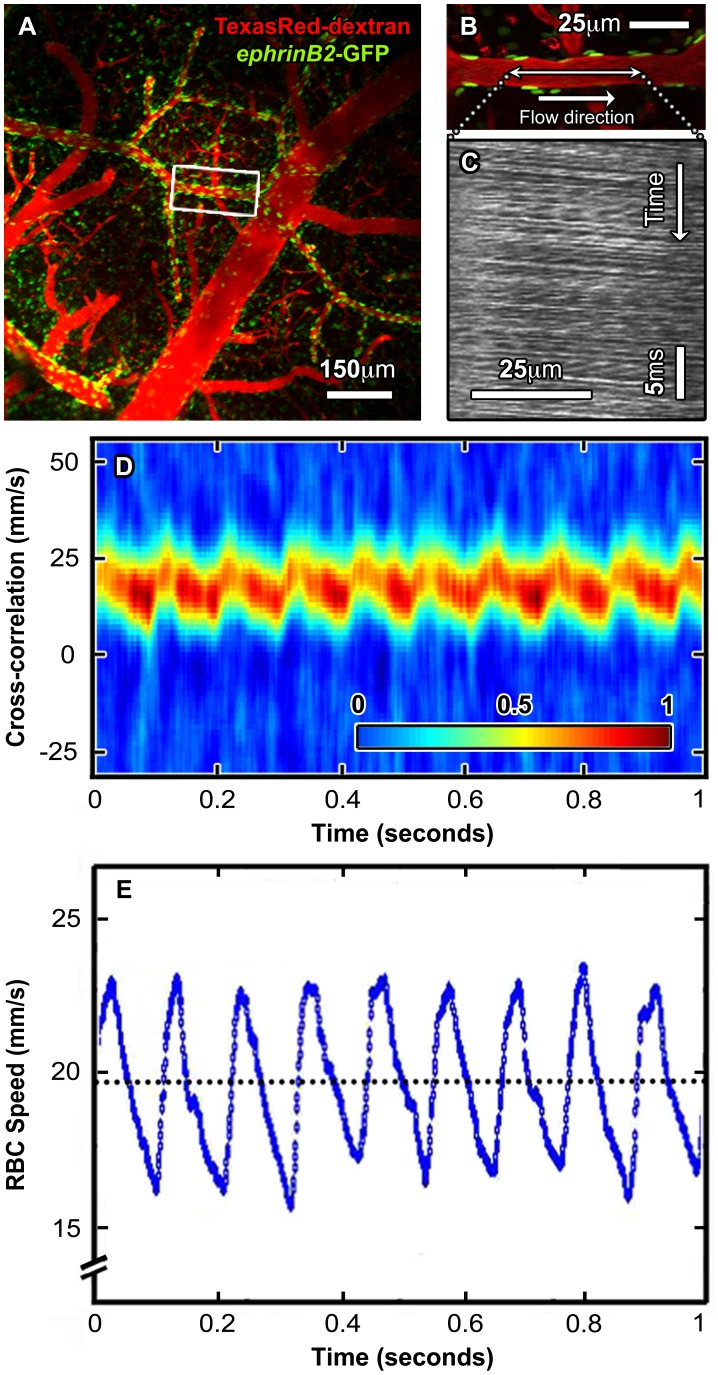
LS-PIV analysis of *in vivo* two-photon line-scan data measured in cortical artery. A , TPLSM projection of cortical vasculature in *ephrin-B2^+/H2B−eGFP^* mouse with the blood plasma labeled by intravenous injection of Texas Red-dextran. *Ephrin-B2^+/H2B−eGFP^* mice express nuclear GFP in arterial endothelial cells, which allows these vessels to be distinguished with cellular resolution. **B**, TPLSM image through the center of an arteriole from the white box in (A). The double-headed arrow indicates the location where line-scans were recorded and the single-headed arrow represents the direction of flow. **C**, Line-scan data from the vessel in (B) where each sequential line-scan appears beneath the one before, forming a space-time image with time increasing from top to bottom. Each dark streak corresponds to a single RBC as it moves along the scan path. **D**, LS-PIV applied between pairs of sequential line-scans from vessel (C). Individual cross-correlation results are oriented along the y-axis with time advancing left to right. Each respective probability distribution is normalized and color-coded according to the inset key. The y-axis is converted from units of distance to units of speed by dividing by the time interval between line-scans. **E**, The final RBC velocity along the scan line is determined from the peak value at each time point in (D). The dotted line represents time-averaged velocity. The temporal resolution of analysis is shown here at 1.3 kHz (1/2 of maximum) to better illustrate individual points.

### LS-PIV Implemented with TPLSM

Figure 1 illustrates the operating principles of LS-PIV. The blood plasma was loaded with fluorescent dye that allowed imaging of perfused vessels. RBCs did not take up dye and could be resolved as dark particles amid the bright background using TPLSM (Figure S2A). Line-scans were recorded along the central axis of vessels within a region-of-interest (ROI) along a straight segment, with a typical length of 10 µm for capillaries and 50 µm or greater for arteries. These ROIs were adjusted by changing the travel distance of the line-scans while maintaining the scan rate at 2.6 kHz for all measurements. Displacement of RBCs between sequential line-scans was determined from their cross-correlation. The shift from the origin to the center of the peak of the cross-correlation is a measure of the spatial shift of RBCs between image frames. RBCs throughout the ROI collectively contributed to the strength of one velocity calculation. To determine the shift with maximum accuracy, the peak was fitted with a Gaussian distribution. The shift in pixels was then converted to microns and velocity was calculated as *v*  =  Δ*x/*Δ*t*_,_ where Δt is the time between line-scans. Importantly, displacement can be determined by this method when RBCs move too quickly to be tracked individually as continuous streaks within the space-time data (Figure S2B)_._ Operations were executed in 32-bit Matlab (Mathworks). Matlab code for LS-PIV is available at: https://sourceforge.net/projects/lspivsupplement/files/with supporting information in Text S1 and Figure S3.

Several techniques are employed to optimize analysis in addition to simple cross-correlation (Table 1). We remove non-moving background prior to cross-correlation by subtracting the time-averaged signal from the line-scan data. Such background is largely a result of absorption or blockage of fluorescence by structures within the light path to the objective. Analysis of the Fourier transform has demonstrated that phase information, not amplitude, is more important in describing an object’s position [43]. We therefore employ symmetric phase-weighted filtering in the Fourier domain, which improves peak determination by reducing side-lobes that frequently appear in the cross-correlation [44]. Additionally, 2.6 kHz temporal resolution is generally more than needed and we can average across several cross-correlation frames to increase the signal-to-noise ratio (SNR).

### Identifying Poor Analysis

The pulsatile velocities from *in vivo* data are an excellent indicator of successful analysis. Erroneous velocity calculations tend to present with catastrophically high or low values, and can be flagged by setting a threshold of standard deviations from a running, windowed mean of analysis. The threshold and window can be adjusted by the user and is included in the Matlab code for LS-PIV (Text S1).

**Figure 3:**
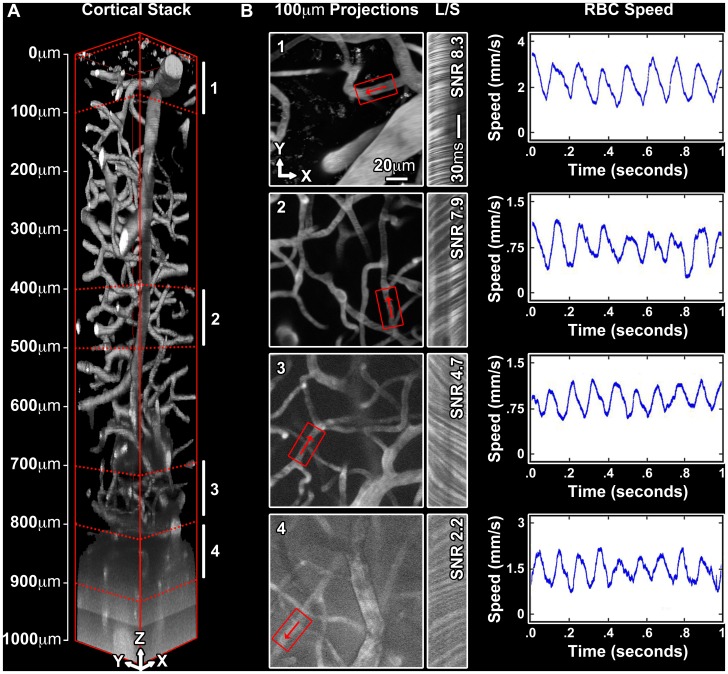
Effects of depth-dependent noise on LS-PIV analysis. A , Three-dimensional rendering of cortical vessels imaged with TPLSM demonstrating depth-dependent decrease in SNR. The blood plasma was labeled with Texas Red-dextran and an image stack over the top 1000 µm was acquired at 1 µm spacing along the z-axis starting from the brain surface. **B**, 100 µm-thick projections of regions 1–4 in panel (A). RBC velocities were measured along the central axis of vessels shown in red boxes, with red arrows representing orientation of flow. The raw line-scan data (L/S) are depicted to the right of each field and labeled with their respective SNR. Corresponding LS-PIV analyses are depicted to the far right.

**Figure 4:**
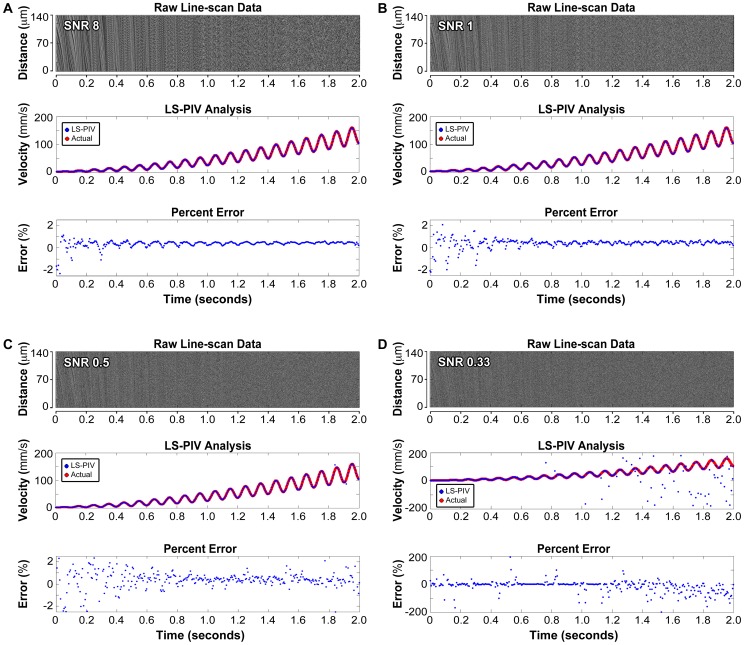
Accuracy of LS-PIV analysis with noise and increasing speed. *Top,* simulation line-scan data with a low level of normally distributed noise with SNR of 8 (**A**), 1 (**B**), 0.5 (**C**), and 0.33 (**D**). *Middle,* LS-PIV analysis of the line-scan data (blue dots). The red line represents actual particle speed. *Bottom,* percent error of LS-PIV compared with actual velocity. Raw simulation data were generated using a randomized distribution of particles that was shifted in known spatial increments over time. The size of each particle was further randomized between 1 to 7 µm to mimic RBCs at different orientations and positions relative to the scan line. Speed was increased over time and oscillated at 10 Hz to simulate mouse heart rate. For comparison, these data are displayed with time along the x-axis aligned with their velocity analyses. Particles initially appear as continuous dark lines within the time-space data but become discontinuous at higher speed, posing a challenge for angle-based analysis. Importantly, LS-PIV performs well at fast velocities where it is difficult to visually track individual particles.

### Execution Speed

In practice, the execution speed of LS-PIV can be very fast. The time-intensive component of analysis is fitting the Gaussian distribution to each cross-correlation peak, a step that can be readily parallelized. Additionally, the maximum temporal resolution of LS-PIV is excessive for most applications (2,600 points per second on our system), and fewer velocity points may be chosen to reduce the number of Gaussian fits and increase speed of analysis. These settings can be adjusted according to the needs of the user (see Text S1 for Matlab code and example data-sets). For example, we found that an *in vivo* data-set with 12,800 line-scans, about a 5 s interval, was completed in 10.9 s when calculating 100 velocity points per second. Analyzing the same data-set with 2,600 velocity points per second was completed in 150.2 s. In both cases, the cross-correlation itself took 2.3 s of the total elapsed time. These analyses were conducted using 12 cores on two 2.6 GHz processors. Additional workers could be implemented to further increase execution speed.

**Figure 5:**
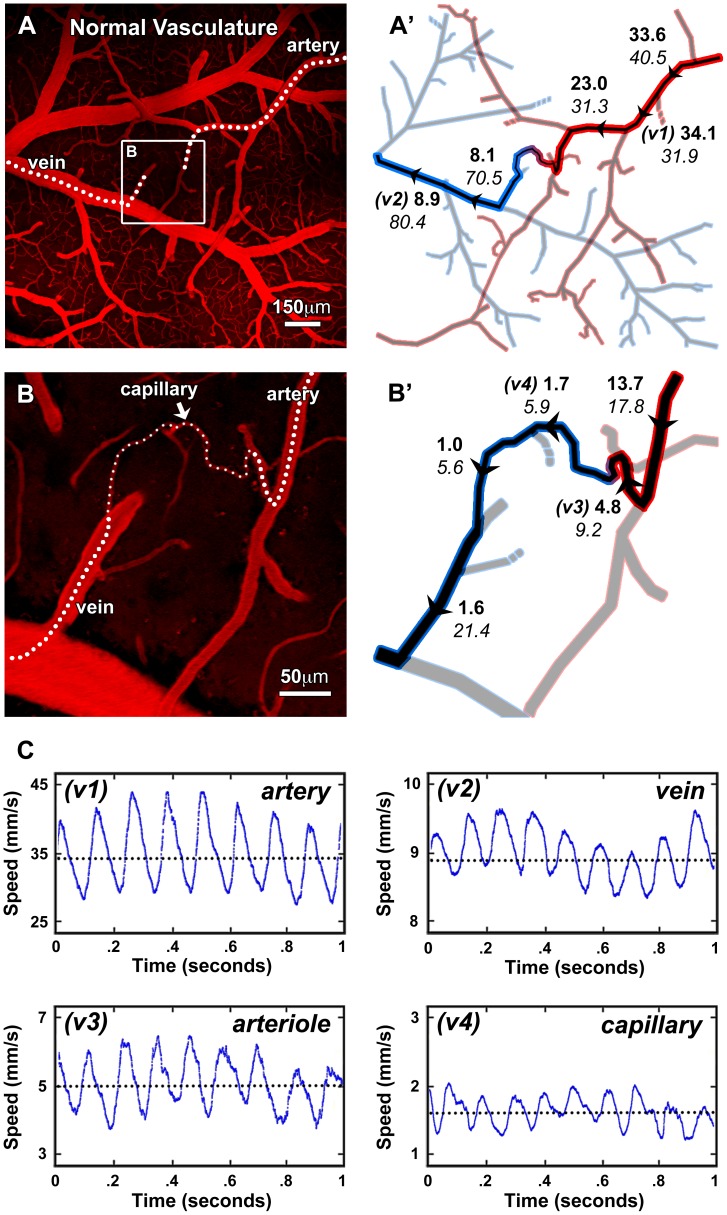
RBC velocities along the normal vessel network in mouse cortex. A , Low-magnification TPLSM image of cortical vasculature in control mouse with the blood plasma labeled by Texas Red-dextran. **A’**, Diagram of the vascular network from image (A) with arteries shown in red and veins in blue. An artery-to-vein pathway is highlighted and labeled with arrowheads to indicate the direction of flow and specific locations of velocity and diameter measurements. Bold numbers represent average center-axis velocity in units of mm/s and italic numbers represent diameter in units of µm corresponding to the adjacent arrowheads. **B**, High magnification TPLSM image from the white box in (A) demonstrating an artery, vein, and interconnecting capillary. Vessels that appear to end abruptly are diving out of the image plane. **B’**, Diagram of the vascular network from image (B). **C**, Representative analyses in a subset of vessels (v1–v4) from panels (A’) and (B’).

**Figure 6:**
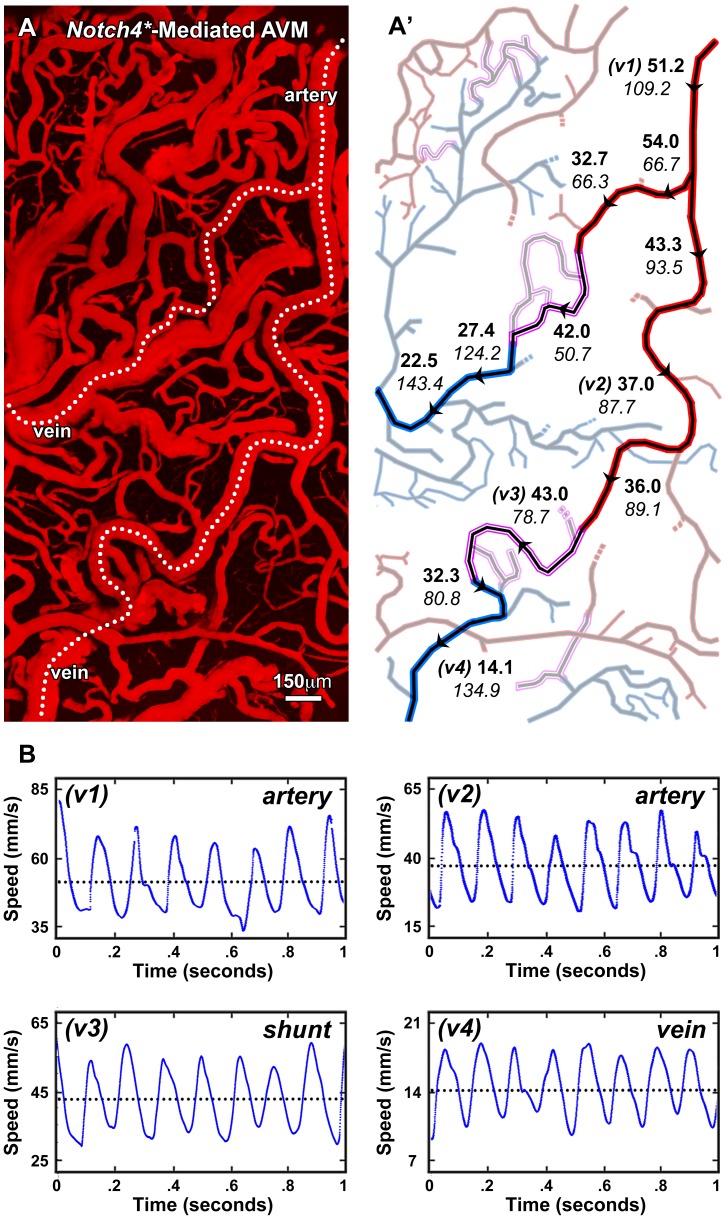
RBC velocities along the vessel network of brain AVMs in mice. **A**, Low-magnification TPLSM image of cortical vasculature in Notch4* mouse model with the blood plasma labeled by Texas Red-dextran. **A’**, Diagram of the vascular network from image (A) with arteries shown in red, veins in blue, and shunts in purple. Artery-to-vein pathways are highlighted and labeled with arrowheads to indicate the direction of flow and specific locations of velocity and diameter measurements. Bold numbers represent average center-axis velocity in units of mm/s and italic numbers represent diameter in units of µm corresponding to the adjacent arrowheads. **B**, Representative analyses in a subset of vessels (v1–v4) from panels (A’).

## Results

### Operational Principles of LS-PIV

We initially found that existing methods for analyzing TPLSM-based data could not quantify RBC velocities in large brain AVMs of Notch4* mice. RBCs often moved too quickly to be tracked as individual streaks in the space-time data (Figure S2A–B). However, we reasoned that many of the same cells were re-imaged in sequential line-scans and could still be used to calculate velocity by cross-correlation analysis. We therefore developed LS-PIV and first tested it in cortical arteries with moderate RBC speeds. Figure 2 shows analysis of line-scan data measured from an arterial segment in a collateral loop. These cerebral collateral vessels were ideal for early tests as they generally harbored lower RBC velocities than similarly sized arteries in arborizing networks. Arteries were identified by their morphology, direction of flow, and endothelial expression of histone-tagged GFP under control of the endogenous promoter for arterial marker *ephrin-B2* [38] (N = 34 vessels in 3 mice, Figure 2A). The blood plasma was labeled with high-molecular-weight Texas Red-dextran and the cortical vasculature was imaged through a cranial window over the right parietal cortex. Image stacks were acquired with 1 µm steps along the optical axis and visualized as average projections (Figure 2A).

Line-scans were recorded at 2.6 kHz along the central axis of vessels and analyzed in a ROI of ∼50 µm corresponding to 3–4 endothelial cells along the adjacent wall (Figure 2B). These data were organized into space-time images with time advancing downward on the ordinate and with distance on the abscissa. Individual RBCs appeared as dark streaks at these moderate velocities (Figure 2C). LS-PIV was applied between every pair of sequential line-scans to produce a running cross-correlation of RBC motion. The initial result was converted to a probability distribution with units of mm/s by dividing by the scan interval (Figure 2D). The final velocity for each time-point was calculated from the most probable value in the corresponding correlation (Figure 2E). These results demonstrated that LS-PIV could analyze RBC velocities without the need to modify existing TPLSM or surgical preparation. Additionally, LS-PIV provided exceptional temporal resolution equal to the line-scan rate (held at 2.6 kHz on our system) and made it easy to see the rapid changes in blood velocity due to heartbeat.

### Performance of LS-PIV with Increasing Noise and Velocity

We sought to validate LS-PIV under demanding conditions using both *in vivo* and simulated data. Previous methods were constrained by their range of analyzable flow speeds or by depth-dependent image quality. We therefore tested the performance of LS-PIV under different SNR and increasing RBC velocity. A major source of image noise in TPLSM results from laser scatter and production of extra-focal fluorescence as the imaging plane is positioned deeper into the specimen [22], [45]. We found that utilizing Texas Red-dextran to label the plasma allowed for deeper imaging depths than fluorescein-dextran (data not shown), likely because of Texas Red-dextran’s large two-photon cross-section and low absorption of red fluorescence by the blood. SNR values were determined from the raw line-scan data in individual vessels using the contrast of RBCs amid the fluorescent plasma. Typical best data was acquired at the surface of the brain and had an SNR of ∼8. High noise data, generally obtained hundreds of micrometers below the surface, had an SNR of ∼2 or less. Figure 3A–B shows increasing noise from progressively deeper imaging in the living brain and LS-PIV analysis down to 850 µm using line-scan data with an SNR of 2.2. To our knowledge, these RBC velocity measurements are the deepest ones recorded with TPLSM without the use of amplified [46] or longer wavelength femtosecond laser pulses [47], and demonstrate the robustness of LS-PIV with well-established imaging parameters.

Next, we examined the true accuracy and range of LS-PIV using simulated raw line-scan data where it was possible to precisely define velocity and noise parameters (Figure 4A–D, upper panels). These data were generated using a randomized distribution of particles that was shifted in known spatial increments over time. The size of each particle was further randomized between 1 to 7 µm to mimic RBCs at different orientations and positions relative to the scan line. Speed was increased with oscillations at 10 Hz to simulate mouse heart rate, and normally distributed noise was added for typical best to very poor data corresponding to a range from SNR of 8 to 0.33. Under these noise conditions, LS-PIV accurately quantified velocities above previously reported values using a ROI of 140 µm (Figure 4A–D, middle panels). LS-PIV analysis is displayed as blue dots and actual velocity is depicted with red lines. LS-PIV demonstrated high fidelity using the data with an SNR of 8, and gradually increasing error with lower SNR values, particularly at very slow and very high velocities (Figure 4A–D, bottom panels). Error manifested as increased scatter and the appearance of distinct outliers. Outliers are a common occurrence in PIV data and can be removed using median filters [19]. We further tested LS-PIV with SNRs ranging from 0.33 to 8 and compared these results with analysis using singular value decomposition (SVD) [20], demonstrating good agreement with the previous method at lower velocities (Figure S4). LS-PIV works well at high velocities and with low signal-to-noise conditions. Additionally, LS-PIV could analyze speeds up to 800 mm/s using an ROI of 426 µm (Figure S5A), demonstrating that LS-PIV is fundamentally capable of analyzing a wide range of velocities with high quality imaging data.

LS-PIV also performed well under capillary-level velocities and in periodic reversals in flow direction (Figure S5B). We analyzed *in vivo* capillary data with LS-PIV and compared the result with SVD, the gold standard at lower velocities (Figure S6). Importantly, we found that RBC displacement in capillaries was often small compared to the pixel resolution of the microscope when two sequential line-scans were compared, which potentially decreased the accuracy of the speed determination. We minimize this inherent error in the raw data by processing non-neighboring line-scans in time (i.e., every line-scan with its Nth neighboring scan) thereby increasing the delay between cross-correlated line-scans and allowing RBC displacement to be large enough to resolve. Another important consideration is that LS-PIV requires RBCs to calculate velocity. While we find that RBCs are usually abundant in most vessels, cerebral capillaries have been observed, at least transiently, to have zero hematocrit [48]. LS-PIV would be unable to quantify velocity in this setting. Thus, LS-PIV has a large dynamic range and enables TPLSM-based quantification of high RBC velocities and slow velocities in individual vessels if hematocrit is non-zero.

### Analysis of RBC Velocities in Normal and AVM Cerebrovasculature

Functional implementation of TPLSM has made it possible to study hemodynamics in living organisms with extremely high detail. However, previous investigations could quantify only the lower range of blood velocities typically found in rodents. We were interested in using LS-PIV to investigate hemodynamics in a mouse model of Notch-mediated AVM and quantifying the fastest reported RBC velocities measured by *in vivo* TPLSM.

We first measured RBC velocities within major artery-to-vein networks overlying the cortex of control mice (N = 28 vessels in 3 mice, Figure 5A–B). The blood plasma was labeled with Texas Red-dextran and arteriovenous identity of vessels was determined by their morphology and flow direction. Line-scans were then recorded along the central axis of contiguous vessel segments between a major branch of the middle cerebral artery and a surface vein draining to the superior sagittal sinus (Figure 5A’–B’). Representative analyses in a subset of these vessels are displayed (Figure 5C). We found that LS-PIV clearly resolved peak arterial velocities up to 44 mm/s in controls (the fastest we measured) as well as slow capillary velocities, which were typically below 2 mm/s. Flow remained pulsatile due to heartbeat in all measured vessels including capillaries and venules, as recently reported [48]. RBC velocity progressively decreased from arteries to capillaries and thereafter increased modestly as flow coalesced into major veins. A slower ∼0.5–2 Hz modulation in RBC speed corresponded with the respiratory rate of the anesthetized animals and was extracted from analysis of arteries or veins (data not shown).

We next combined *in vivo* TPLSM with a mouse model of Notch-mediated AVMs [37] and directly investigated hemodynamics within the aberrant artery-to-vein topology in the brain. The most important determinant of hemodynamics within an AVM is its resistance to blood flow. This is largely dependent on the narrowest cross-sectional point along the flow path, where increases in diameter result in dramatic loss of resistance and from which many pathophysiological phenomena of AVMs are speculated to follow [13]. We therefore identified individual arteriovenous shunts and measured center axis velocities in contiguous vessel segments in both the up- and down-stream pathways (N = 42 vessels in 3 mice, Figure 6A). These vessels were tortuous and dramatically enlarged. Representative analyses in a subset of these vessels are displayed in Figure 6B. We found that LS-PIV clearly resolved peak RBC velocities up to 84 mm/s (the fastest we measured) and that velocities were elevated and markedly pulsatile within all compartments of the vasculature. Interestingly, velocities often spiked within AV shunts (Figure 6B, purple segments) compared with up- and down-stream segments. These shunts exhibited laminar flow when we examined their average, peak systolic, and end-diastolic spatial flow profiles (Figure S7). Average RBC velocities progressively decreased toward and from the AV shunts within the measurable field (Figure 6A’), without increasing again in the coalescing veins. These results highlight the impact that shunts have on hemodynamics within the arteriovenous network and demonstrate the utility of LS-PIV in studying a wide range of flow speeds with microscopic resolution.

## Discussion

Advances in genetic labeling techniques and functional imaging approaches have helped establish TPLSM and confocal microscopy as a powerful, noninvasive ways to study vascular biology in whole-tissue explants and living organisms [22], [23], [24], [30]. Hemodynamics have been investigated with capillary-level resolution in brain [20], kidney [49], and tumor [50], [51]. However, the adult mouse harbors far faster RBC velocities than can be analyzed by previous methods for analyzing TPLSM- or confocal-based data. LS-PIV enables analysis of a wide range of RBC velocities by easy integration with well-established *in vivo* imaging and line-scan acquisition approaches. We demonstrated the utility of this method by robustly quantifying blood velocities in a mouse model of brain AVM. To our knowledge, these are the fastest RBC velocities analyzed using multi-photon or confocal microscopy. Our simulations indicate that LS-PIV may also analyze a wider range of velocities and be useful in studying high-flow vessels in mice.

An important feature of LS-PIV is its ability to resolve the spatial profile of simple flows within individual vessels by using multiple line-scans positioned near each other. This is enabled by the diffraction-limited resolution of TPLSM, where an image voxel on our system is about ∼0.4 µm in the image (‘xy’) plane and ∼1.5 µm along the optical (‘z’) axis. Importantly, LS-PIV determines the component of RBC velocity along the scan line itself but does not inherently determine the orientation of the streamline. Therefore in this study, we focused on relatively strait vessel segments to minimize flow complexity. As a proof-of-principle, we measured the flow in a control artery and a Notch-mediated AV shunt by acquiring multiple axial line-scans spaced every few micrometers across the diameters of their lumens (Figure S7). Complete profile measurements took roughly 10–15 minutes, which could be drastically shortened by using pre-patterned line-scan arrays and reducing the number of scans per vessel. Additionally, multiple line-scans can be acquired at varying angles for the same region, where the orientation with the highest velocity would most accurately represent the streamline. Such systematic approaches, combined with the temporal resolution of LS-PIV, would also allow for quantification of pulsatile dynamics including the spatial profiles of peak systolic and end-diastolic flow (Figure S7). These highly resolved measurements have the potential to address long-standing questions on how local hemodynamic forces influence endothelium and vascular development *in vivo*. For example, it is established from *in vitro* studies that hemodynamic shear stress regulates endothelial cell proliferation, shape, alignment, and gene expression [52], [53], [54], [55]. Arterial and pulsatile waveforms regulate ‘athero-prone’ and ‘athero-protective’ genes [56], [57] suggesting that peak systolic or end-diastolic shear forces may be important stimuli for flow-responsive programs. To our knowledge, there is currently no method to directly correlate high shear rates from fast blood flow with cellular-level dynamics and vascular remodeling in animals. Application of LS-PIV in a systematic fashion will shed light on the role of high pulsatile shear in vessel development, maintenance, and disease.

LS-PIV may be combined with a growing number of transgenic approaches to study the relationship between hemodynamics, cellular behavior, and biochemical signaling *in vivo*. For example, *ephrin-B2^+/H2B−eGFP^* mice [38] allow visualization of arterial endothelium with cellular resolution by TPLSM (Figure 2A–B) which could be used to track these cells over time. Tissue-specific and temporally inducible methods may be used to test the role of key biochemical constituents on vessel maintenance or malformation within various hemodynamics environments. We use the *Tie2-tTA* and TRE-Notch4* mice [36], [37] to express Notch4* in endothelium of post-natal mice. Combining LS-PIV with such genetic approaches will be helpful in studying the role of hemodynamic cues in developmental programs.

An important limitation of TPLSM has traditionally been its slow scanning rate along the optical axis, which has been achieved by translating the position of the sample relative to the objective lens with a stepper motor. While sufficient for three-dimensional imaging, this approach has considerable inertial constraints that prevent rapid laser scanning oblique to the image plane. Two previous approaches have attempted to obviate the need for rapid scanning along the optical axis to quantify blood flow in three-dimensions: residence time line scanning (RTLS) velocimetry [33] and Doppler optical coherence tomography (DOCT) [58]. RTLS velocimetry extrapolates RBC velocities at all orientations from two-photon line-scans collected in the ‘xy’ plane. This analysis utilizes the angle between the scan line and vessel axis, and the residence time of the RBCs as they pass the scan region. However, RTLS will not work to calculate high velocities at oblique angles because RBCs pass too quickly through the scan line to be imaged more than once. Alternatively, DOCT can quantify blood flow simultaneously across many ascending and descending vessels by measuring the Doppler shift of light scattered off moving RBCs. Though a powerful approach for measuring network flow in an unbiased fashion, DOCT is currently applicable to speeds under 10 mm/s, and in larger vessels rather than capillaries [59]. Additional methods are required to analyze high RBC velocities with microscopic resolution in three-dimensions. Recently, rapid three-dimensional scanning has been achieved using acousto-optic deflectors (AOD) to steer the laser beam without moving the animal or the objective lens [60]. This approach reportedly has an effective scan range of 50 µm at a rate of 3–10 kHz along the optical axis using a high numerical aperture objective, which would be sufficient for direct line-scan measurements of a wide range of RBC velocities. Implementation of three-dimensional AOD scanning with LS-PIV would enable analysis of high blood velocities in all orientations.

Although LS-PIV was able to analyze our fastest *in vivo* data (peak velocities up to 84 mm/s), vessels that harbor higher velocities are too deep in the intact animal for TPLSM. Protocols are actively being developed to allow *in vivo* microscopy in deep organs such as the lung, ovary, and spinal cord [61], [62], [63], and similar approaches will enable exciting new studies of blood flow in visceral vessels. We used raw simulation data to test higher speeds up to 800 mm/s, demonstrating that LS-PIV fundamentally can work with faster velocities. However, an important consideration is that fast *in vivo* data may develop artifacts associated with non-ideal imaging conditions. For example, at very high speeds, RBC displacement may be significant relative to the scanning speed of the laser focus. The patterns in the line-scan images could be stretched or compressed depending on the direction of blood flow relative to the scan. The effect of this artifact on velocity calculation may be mitigated by the use of bi-directional laser scanning (as used in this study) where alternating sweeps of the laser inverts the relative direction of the blood flow and line-scan. Improvement in faster laser-scanning technologies will also reduce this artifact [60]. Additionally, pulsatile motion associated with large arteries may introduce distortions in the raw data. Such artifact may be minimized by gating data acquisition with cardiac and respiratory activity [48], [64]. Future work with faster *in vivo* data will help determine which artifacts are limiting and help guide further improvements in data acquisition and analysis.

In conclusion, TPLSM has been the tool of choice for studying hemodynamics with capillary-level resolution and large penetration depths, but has traditionally been limited to studying moderate blood flows. We have developed LS-PIV, which may be easily integrated with well-established *in vivo* imaging and line-scan data acquisition approaches, in order to quantify higher blood velocities with TPLSM or confocal microscopy. LS-PIV performed robustly with noisy data in vessels as deep as 850 µm below the brain surface and with very high accuracy as validated with simulation data. As a proof of principle, we investigated blood velocities within the vascular network of brain AVM in mice. LS-PIV is uniquely suited to study high RBC velocities with optical resolution deep in living animals. Partnered with transgenic mice with cell-specific fluorescent reporters or with exogenous dyes, LS-PIV will enable the direct *in vivo* correlation of cellular, biochemical, and hemodynamic cues in high flow processes such as atherogenesis, arteriogenesis, and AVMs.

## Supporting Information

Figure S1 **Schematic of the two-photon laser scanning microscope.** Images and line-scan data were obtained using a locally constructed TPLSM. 100 femtosecond, 80 MHz pulses were generated by a Titanium:Sapphire laser oscillator (Mai Tai HP; Newport Spectra-Physics) and passed through a dispersion compensator (DeepSee; Newport Spectra-Physics). Laser intensity was controlled by rotating a λ/2 wave-plate relative to a polarizer, and the attenuated beam was directed onto the microscope platform. The laser pulses were raster-scanned by galvonometric mirrors and relay-imaged to the back aperture of a 1.0 NA water-immersion objective (Zeiss). Two-photon excited fluorescence was collected by the same objective, reflected by a dichroic mirror (700 nm long pass, dichroic 1), split into red or green channels by a second dichroic mirror (560 nm long pass, dichroic 2), spectrally cleaned with additional band-pass filters (Chroma), and relayed to photomultiplier tubes (PMT; H7422P-40MOD; Hamamatsu). The anesthetized mouse was immobilized with a stereotax (myNeurolab.com) and placed on a three-dimensional translation stage (11SI70521 Rev. 01; Newport) that allowed precise positioning of the mouse relative to the focus of the microscope. MPScan 1.0 software was used to control the microscope and record image data.
(TIF)

Figure S2 **Loss of RBC continuity in space-time data when high velocities are measured with line-scans. A,** Schematic of line-scan data of slow to moderate RBC velocities. Each sequential line-scan appears beneath the previous one, with time advancing from top to bottom. RBCs appear as continuous diagonal streaks in the space-time image when RBC velocities are relatively slow compared to the line-scan rate. Previous analytical methods have determined velocities by calculating the inverse slope of the streaks. **B,** Schematic of line-scan data of fast RBC velocities. RBCs may appear as discontinuous streaks or as just a few dark spots in the space-time image. The large number of RBCs confounds identification of individual streaks.
(TIF)

Figure S3 **Example of output of analysis running Matlab code for LS-PIV.** Completed analysis with Matlab code depicts the raw data with time aligned along the x axis, a composite of the cross-correlations, and Gaussian-fitted displacements. Note that these results are reported in units of ‘pixels’ for both displacement and time, where the spatial and temporal values of each pixel will depend on the experimental setup. The displacements should be divided by the time of one scan interval to convert to true velocity.
(TIF)

Figure S4 **Effects of noise on performance of LS-PIV and SVD.** Analyses of simulation line-scan data using LS-PIV and previously published SVD. Data had increasing velocity and SNRs ranging from 0.33 to 8. Blue and red dots correspond to LS-PIV and SVD analysis, respectively. Analysis parameters were matched between LS-PIV and SVD using a 100 µm-long region of interest and the same processing time. Agreement between these methods was best at lower velocities and diverged with increasing speed.
(TIF)

Figure S5 **Performance of LS-PIV with high velocities and flow reversal. A**, LS-PIV analysis of simulation line-scan data with velocity increasing to 800 mm/s. Actual particle velocity is represented by the blue line (SNR of infinity). Analysis was performed on data with an SNR of 8, within a 426 µm-long region of interest, and displayed as red dots. **B,** LS-PIV analysis on simulation line-scan data with increasing velocity and reversal of flow. Actual particle velocity is represented by the blue line (SNR of infinity). Analysis was performed on data with an SNR of 8 and displayed as red dots.
(TIF)

Figure S6 **Analyses of** ***in vivo*** **capillary data by LS-PIV and SVD. A,** Analyses of *in vivo* line-scan data using LS-PIV (blue line) demonstrating good agreement with SVD (red line), the gold standard, on capillary flow. In this example, cross-correlations for LS-PIV were conducted between every line-scan and its 5^th^ neighboring scan. **B,** Plot of the difference in velocities calculated by SVD and LS-PIV analysis, corresponding in time with panel (A). The difference corresponds to 4.9% of the standard deviation of the average velocity determined by LS-PIV.
(TIF)

Figure S7 **Cross-sectional flow profiles in control artery and AV shunt. A**, RBC speed as a function of transverse position in a control artery. **B,** RBC speed as a function of transverse position in an AV shunt. Maximum, mean, and minimum RBC speeds were calculated from pulsatile velocities across 5 s scans. Five separate datasets were analyzed at each position in the vessel to determine the final velocities and their standard deviations (vertical error bars). Measurements were acquired serially along the vessel diameter over the span of ∼15 minutes. Vertical dotted lines represent the position of the vessel walls. Interestingly, measured RBC velocities did not go to zero at the wall, likely because of the cells’ finite size and inability to move closer to the boundary than half their width. Positional error was estimated additively from the optical resolution, translation stage jitter, and motion artifact of the animals (horizontal error bars).
(TIF)

Text S1 **Example Line-scan Data and LS-PIV Matlab Implementation.**
(PDF)

## References

[1] Carmeliet P (2003). Angiogenesis in health and disease.. Nature Med.

[2] Jain RK (2003). Molecular regulation of vessel maturation.. Nature Med.

[3] Adams RH, Alitalo K (2007). Molecular regulation of angiogenesis and lymphangiogenesis.. Nature Rev Mol Cell Biol.

[4] Murray CD (1926). The Physiological Principle of Minimum Work Applied to the Angle of Branching of Arteries.. J Gen Physiol.

[5] Murray CD (1926). The Physiological Principle of Minimum Work: I. The Vascular System and the Cost of Blood Volume.. Proc Natl Acad of Sci U S A.

[6] Kurz H (2000). Physiology of angiogenesis.. J Neurooncol.

[7] le Noble F, Moyon D, Pardanaud L, Yuan L, Djonov V (2004). Flow regulates arterial-venous differentiation in the chick embryo yolk sac.. Development.

[8] Conway SJ, Kruzynska-Frejtag A, Kneer PL, Machnicki M, Koushik SV (2003). What cardiovascular defect does my prenatal mouse mutant have, and why?. Genesis.

[9] Huang C, Sheikh F, Hollander M, Cai C, Becker D (2003). Embryonic atrial function is essential for mouse embryogenesis, cardiac morphogenesis and angiogenesis.. Development.

[10] Lucitti JL, Jones EA, Huang C, Chen J, Fraser SE (2007). Vascular remodeling of the mouse yolk sac requires hemodynamic force.. Development.

[11] Herbert SP, Huisken J, Kim TN, Feldman ME, Houseman BT (2009). Arterial-venous segregation by selective cell sprouting: an alternative mode of blood vessel formation.. Science.

[12] Wang HU, Chen ZF, Anderson DJ (1998). Molecular distinction and angiogenic interaction between embryonic arteries and veins revealed by ephrin-B2 and its receptor Eph-B4.. Cell.

[13] Morgan M, Winder M (2001). Haemodynamics of arteriovenous malformations of the brain and consequences of resection: a review.. Journal Clin Neurosci.

[14] Corti P, Young S, Chen CY, Patrick MJ, Rochon ER (2011). Interaction between alk1 and blood flow in the development of arteriovenous malformations.. Development.

[15] Rosenblum WI, El-Sabban F (1981). Measurement of red cell velocity with a two-slit technique and cross-correlation: use of reflected light, and either regulated dc or unregulated ac power supplies.. Microvasc Res.

[16] Wayland H, Johnson PC (1967). Erythrocyte velocity measurement in microvessels by a two-slit photometric method.. J Appl Physiol.

[17] Tyml K, Sherebrin MH (1980). A method for on-line measurements of red cell velocity in microvessels using computerized frame-by-frame analysis of television images.. Microvasc Res.

[18] Gulati S, Muller SJ, Liepmann D (2008). Direct measurements of viscoelastic flows of DNA in a 2:1 abrupt planar micro-contraction.. J Non-Newtonian Fluid Mech.

[19] Willert CE, Gharib M (1991). Digital particle image velocimetry.. Exp Fluids.

[20] Kleinfeld D, Mitra PP, Helmchen F, Denk W (1998). Fluctuations and stimulus-induced changes in blood flow observed in individual capillaries in layers 2 through 4 of rat neocortex.. Proc Natl Acad of Sci U S A.

[21] Shih AY, Driscoll JD, Drew PJ, Nishimura N, Schaffer CB (2012). Two-photon microscopy as a tool to study blood flow and neurovascular coupling in the rodent brain.. J Cereb Blood Flow Metab [epub ahead of print, DOI: 10.1038/jcbfm.2011.196].

[22] Helmchen F, Denk W (2005). Deep tissue two-photon microscopy.. Nat Methods.

[23] Rubart M (2004). Two-photon microscopy of cells and tissue.. Circ Res.

[24] Zipfel WR, Williams RM, Webb WW (2003). Nonlinear magic: multiphoton microscopy in the biosciences.. Nat Biotechnol.

[25] Shih AY, Friedman B, Drew PJ, Tsai PS, Lyden PD (2009). Active dilation of penetrating arterioles restores red blood cell flux to penumbral neocortex after focal stroke.. J Cereb Blood Flow Metab.

[26] Parzy E, Miraux S, Franconi JM, Thiaudiere E (2009). In vivo quantification of blood velocity in mouse carotid and pulmonary arteries by ECG-triggered 3D time-resolved magnetic resonance angiography.. NMR Biomed.

[27] Amirbekian S, Long RC, Consolini MA, Suo J, Willett NJ (2009). In vivo assessment of blood flow patterns in abdominal aorta of mice with MRI: implications for AAA localization.. Am J Physiol Heart Circ Physiol.

[28] Huo Y, Guo X, Kassab GS (2008). The flow field along the entire length of mouse aorta and primary branches.. Ann Biomed Eng.

[29] Drew PJ, Blinder P, Cauwenberghs G, Shih AY, Kleinfeld D (2010). Rapid determination of particle velocity from space-time images using the Radon transform.. J Comput Neurosci.

[30] Jones EA, Baron MH, Fraser SE, Dickinson ME (2004). Measuring hemodynamic changes during mammalian development.. Am J Physiol Heart Circ Physiol.

[31] Nishimura N, Schaffer CB, Friedman B, Tsai PS, Lyden PD (2006). Targeted insult to subsurface cortical blood vessels using ultrashort laser pulses: three models of stroke.. Nat Methods.

[32] Schaffer CB, Friedman B, Nishimura N, Schroeder LF, Tsai PS (2006). Two-photon imaging of cortical surface microvessels reveals a robust redistribution in blood flow after vascular occlusion.. PLoS Bio.

[33] Kamoun WS, Chae SS, Lacorre DA, Tyrrell JA, Mitre M (2010). Simultaneous measurement of RBC velocity, flux, hematocrit and shear rate in vascular networks.. Nature Methods.

[34] Ellis CG, Ellsworth ML, Pittman RN, Burgess WL (1992). Application of image analysis for evaluation of red blood cell dynamics in capillaries.. Microvasc Res.

[35] Japee SA, Pittman RN, Ellis CG (2005). Automated method for tracking individual red blood cells within capillaries to compute velocity and oxygen saturation.. Microcirculation.

[36] Carlson TR, Yan Y, Wu X, Lam MT, Tang GL (2005). Endothelial expression of constitutively active Notch4 elicits reversible arteriovenous malformations in adult mice.. Proc Natl Acad Sci U S A.

[37] Murphy PA, Lam MT, Wu X, Kim TN, Vartanian SM (2008). Endothelial Notch4 signaling induces hallmarks of brain arteriovenous malformations in mice.. Proc Natl Acad Sci U S A.

[38] Davy A, Bush JO, Soriano P (2006). Inhibition of gap junction communication at ectopic Eph/ephrin boundaries underlies craniofrontonasal syndrome.. PLoS Biol.

[39] Kleinfeld D, Denk W (2000). Two-photon imaging of neocortical micro-circulation.. In: Yuste R, Lanni F, Konnerth A, eds Imaging neurons: A laboratory manual Cold Spring Harbor, New York: Cold Spring Harbor Laboratory Press 231–2315..

[40] Hornig S, Christoph B, Gafe A, Wotschadlo J, Liebert T (2008). Biocompatible fluorescent nanoparticles for pH-sensoring.. Soft Matter.

[41] Nguyen QT, Tsai PS, Kleinfeld D (2006). MPScope: a versatile software suite for multiphoton microscopy.. J of Neurosci Methods.

[42] Nishimura N, Rosidi NL, Iadecola C, Schaffer CB (2010). Limitations of collateral flow after occlusion of a single cortical penetrating arteriole.. J Cereb Blood Flow Metab.

[43] Oppenheim AV, Lim JS (1981). The importance of phase in signals.. Proc IEEE.

[44] Wernet MP (2005). Symmetric phase only filtering: a new paradigm for DPIV data processing.. Meas Sci and Technol.

[45] Oheim M, Beaurepaire E, Chaigneau E, Mertz J, Charpak S (2001). Two-photon microscopy in brain tissue: parameters influencing the imaging depth.. J Neurosci Methods.

[46] Theer P, Hasan MT, Denk W (2003). Two-photon imaging to a depth of 1000 microm in living brains by use of a Ti:Al2O3 regenerative amplifier.. Opt Lett.

[47] Kobat D, Durst ME, Nishimura N, Wong AW, Schaffer CB (2009). Deep tissue multiphoton microscopy using longer wavelength excitation.. Opt Express.

[48] Santisakultarm TP, Cornelius NR, Nishimura N, Schafer AI, Silver RT (2012). In vivo two-photon excited fluorescence microscopy reveals cardiac- and respiration-dependent pulsatile blood flow in cortical blood vessels in mice.. Am J Physiol Heart Circ Physiol.

[49] Kang JJ, Toma I, Sipos A, McCulloch F, Peti-Peterdi J (2006). Quantitative imaging of basic functions in renal (patho)physiology.. Am Journal Physiol Renal Physiol.

[50] Brown EB, Campbell RB, Tsuzuki Y, Xu L, Carmeliet P (2001). In vivo measurement of gene expression, angiogenesis and physiological function in tumors using multiphoton laser scanning microscopy.. Nat Medicine.

[51] Kashiwagi S, Tsukada K, Xu L, Miyazaki J, Kozin SV (2008). Perivascular nitric oxide gradients normalize tumor vasculature.. Nat Medicine.

[52] Chien S (2007). Mechanotransduction and endothelial cell homeostasis: the wisdom of the cell.. Am J Physiol Heart Circ Physiol.

[53] Tzima E, Irani-Tehrani M, Kiosses WB, Dejana E, Schultz DA (2005). A mechanosensory complex that mediates the endothelial cell response to fluid shear stress.. Nature.

[54] Li YS, Haga JH, Chien S (2005). Molecular basis of the effects of shear stress on vascular endothelial cells.. J Biomech.

[55] del Alamo JC, Norwich GN, Li YS, Lasheras JC, Chien S (2008). Anisotropic rheology and directional mechanotransduction in vascular endothelial cells.. Proc Natl Acad Sci U S A.

[56] Dai G, Kaazempur-Mofrad MR, Natarajan S, Zhang Y, Vaughn S (2004). Distinct endothelial phenotypes evoked by arterial waveforms derived from atherosclerosis-susceptible and -resistant regions of human vasculature.. Proc Natl Acad Sci U S A.

[57] Buschmann I, Pries A, Styp-Rekowska B, Hillmeister P, Loufrani L (2010). Pulsatile shear and Gja5 modulate arterial identity and remodeling events during flow-driven arteriogenesis.. Development.

[58] Srinivasan VJ, Atochin DN, Radhakrishnan H, Jiang JY, Ruvinskaya S (2011). Optical coherence tomography for the quantitative study of cerebrovascular physiology.. J Cereb Blood Flow Metab.

[59] Santisakultarm TP, Schaffer CB (2011). Optically quantified cerebral blood flow.. J Cereb Blood Flow Metab.

[60] Duemani Reddy G, Kelleher K, Fink R, Saggau P (2008). Three-dimensional random access multiphoton microscopy for functional imaging of neuronal activity.. Nat Neurosci.

[61] Looney MR, Thornton EE, Sen D, Lamm WJ, Glenny RW (2011). Stabilized imaging of immune surveillance in the mouse lung.. Nat Methods.

[62] Farrar MJ, Bernstein IM, Schlafer DH, Cleland TA, Fetcho JR (2012). Chronic in vivo imaging in the mouse spinal cord using an implanted chamber.. Nat Methods.

[63] Williams RM, Flesken-Nikitin A, Ellenson LH, Connolly DC, Hamilton TC (2010). Strategies for high-resolution imaging of epithelial ovarian cancer by laparoscopic nonlinear microscopy.. Transl Oncol.

[64] Megens RT, Reitsma S, Prinzen L, oude Egbrink MG, Engels W (2010). In vivo high-resolution structural imaging of large arteries in small rodents using two-photon laser scanning microscopy.. J Biomed Opt.

