# Supplementary figures and images for: Line-Scanning Particle Image Velocimetry: An Optical Approach for Quantifying a Wide Range of Blood Flow Speeds in Live Animals
Source: PLoS One. 2012 Jun 26;7(6):e38590. doi: 10.1371/journal.pone.0038590 (PMC3383695; doi:10.1371/journal.pone.0038590)

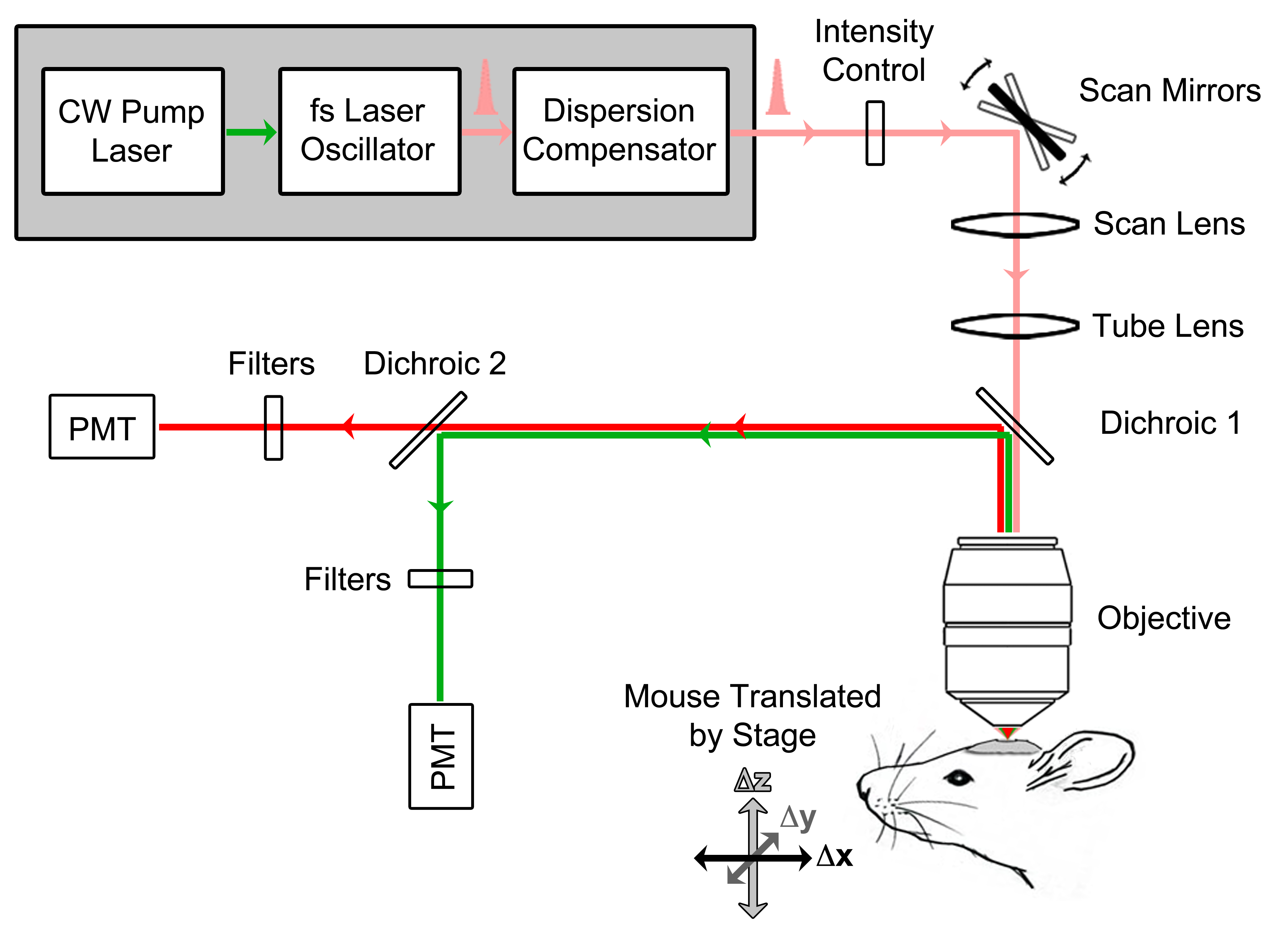

Supplement: Figure S1 — Schematic of the two-photon laser scanning microscope. Images and line-scan data were obtained using a locally constructed TPLSM. 100 femtosecond, 80 MHz pulses were generated by a Titanium:Sapphire laser oscillator (Mai Tai HP; Newport Spectra-Physics) and passed through a dispersion compensator (DeepSee; Newport Spectra-Physics). Laser intensity was controlled by rotating a λ/2 wave-plate relative to a polarizer, and the attenuated beam was directed onto the microscope platform. The laser pulses were raster-scanned by galvonometric mirrors and relay-imaged to the back aperture of a 1.0 NA water-immersion objective (Zeiss). Two-photon excited fluorescence was collected by the same objective, reflected by a dichroic mirror (700 nm long pass, dichroic 1), split into red or green channels by a second dichroic mirror (560 nm long pass, dichroic 2), spectrally cleaned with additional band-pass filters (Chroma), and relayed to photomultiplier tubes (PMT; H7422P-40MOD; Hamamatsu). The anesthetized mouse was immobilized with a stereotax (myNeurolab.com) and placed on a three-dimensional translation stage (11SI70521 Rev. 01; Newport) that allowed precise positioning of the mouse relative to the focus of the microscope. MPScan 1.0 software was used to control the microscope and record image data. (TIF) [file pone.0038590.s001.tif]

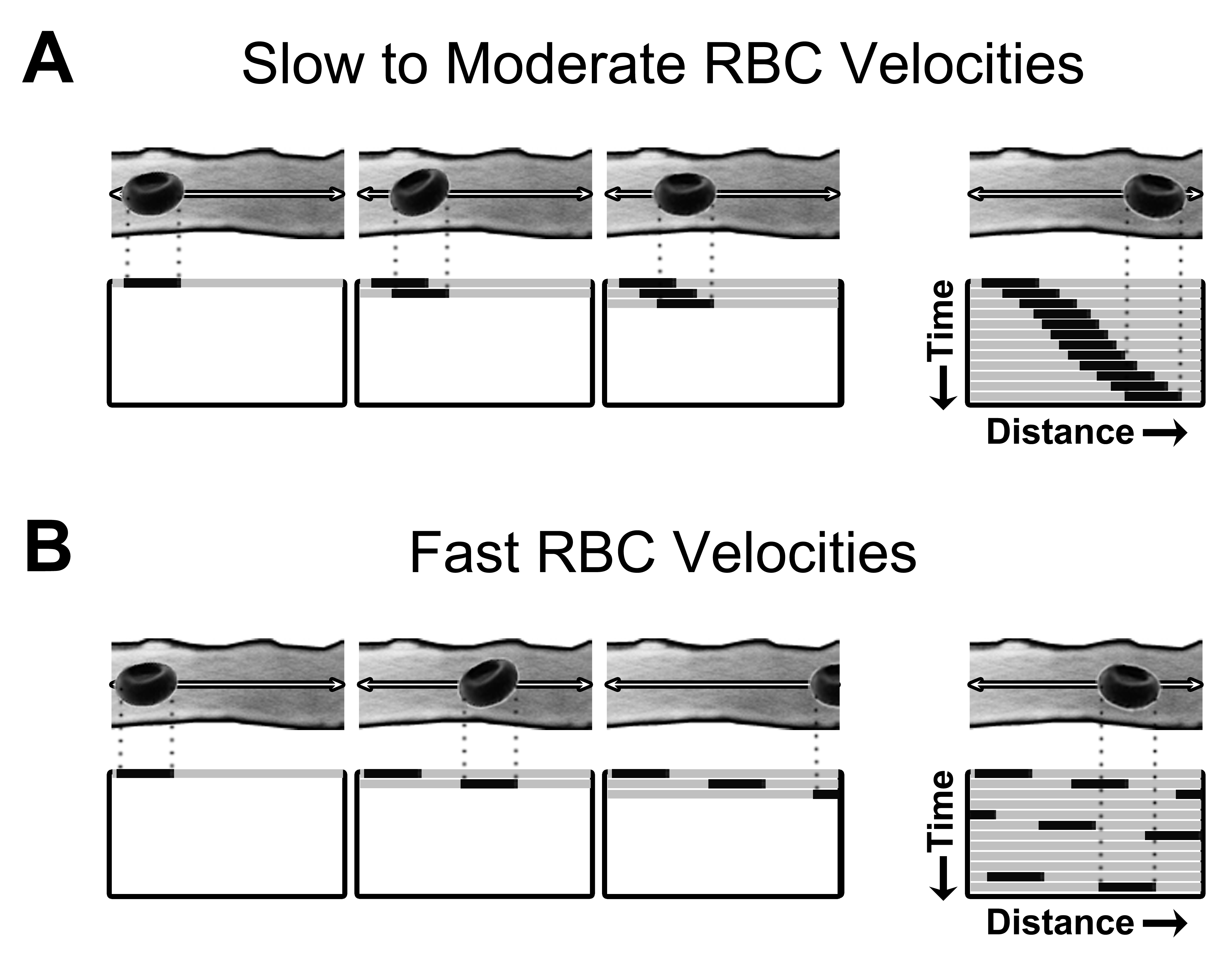

Supplement: Figure S2 — Loss of RBC continuity in space-time data when high velocities are measured with line-scans. A, Schematic of line-scan data of slow to moderate RBC velocities. Each sequential line-scan appears beneath the previous one, with time advancing from top to bottom. RBCs appear as continuous diagonal streaks in the space-time image when RBC velocities are relatively slow compared to the line-scan rate. Previous analytical methods have determined velocities by calculating the inverse slope of the streaks. B, Schematic of line-scan data of fast RBC velocities. RBCs may appear as discontinuous streaks or as just a few dark spots in the space-time image. The large number of RBCs confounds identification of individual streaks. (TIF) [file pone.0038590.s002.tif]

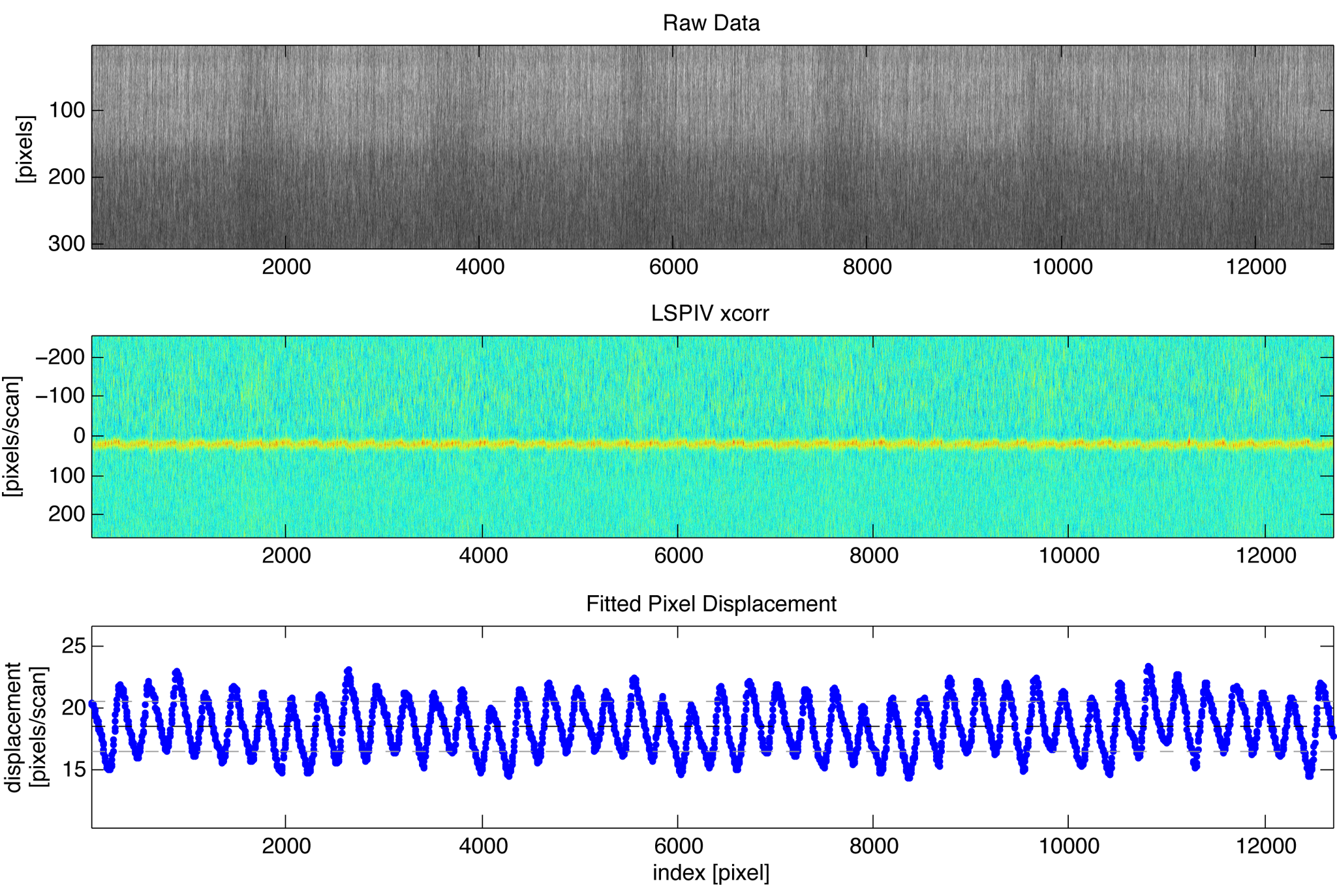

Supplement: Figure S3 — Example of output of analysis running Matlab code for LS-PIV. Completed analysis with Matlab code depicts the raw data with time aligned along the x axis, a composite of the cross-correlations, and Gaussian-fitted displacements. Note that these results are reported in units of ‘pixels’ for both displacement and time, where the spatial and temporal values of each pixel will depend on the experimental setup. The displacements should be divided by the time of one scan interval to convert to true velocity. (TIF) [file pone.0038590.s003.tif]

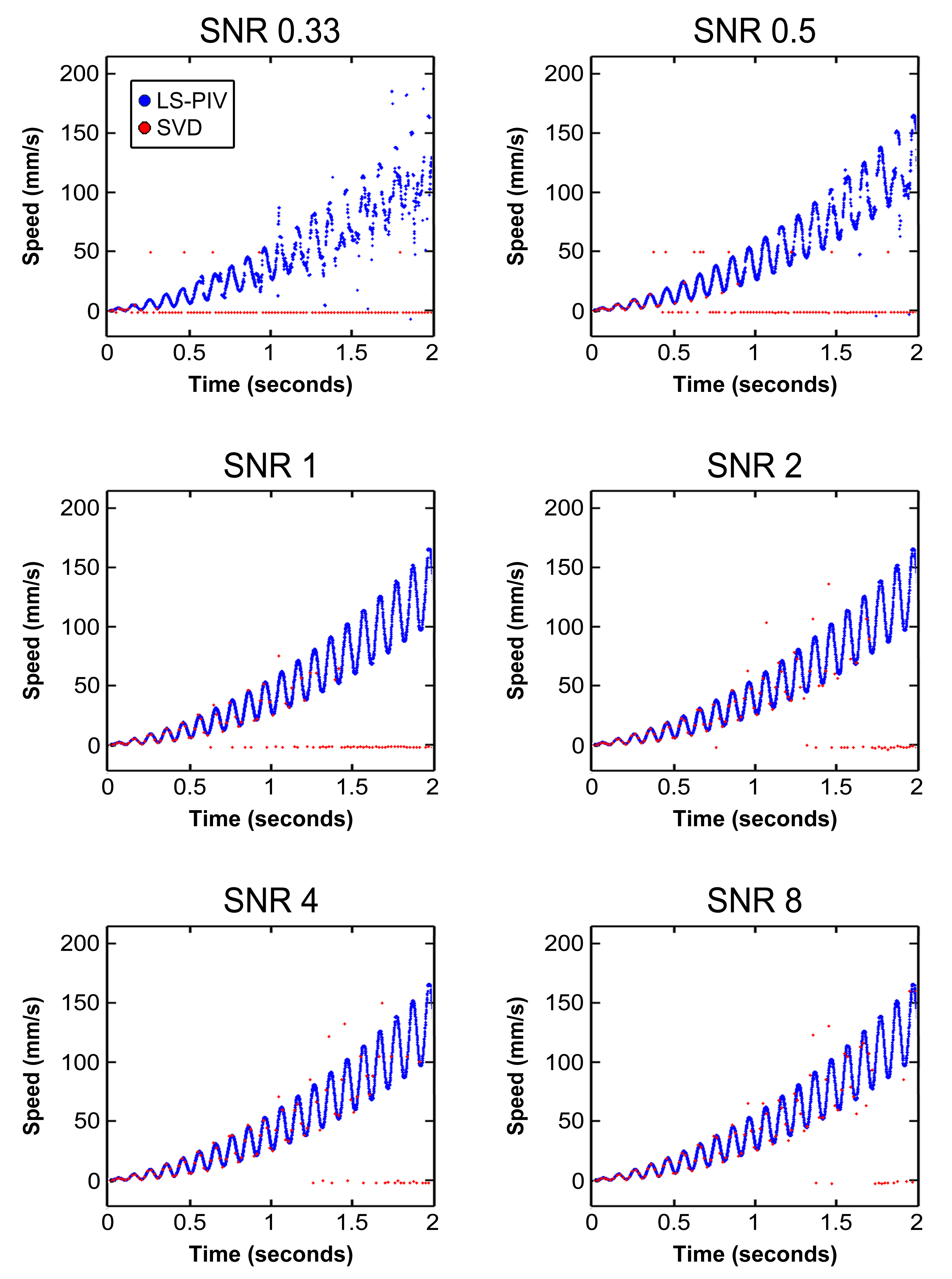

Supplement: Figure S4 — Effects of noise on performance of LS-PIV and SVD. Analyses of simulation line-scan data using LS-PIV and previously published SVD. Data had increasing velocity and SNRs ranging from 0.33 to 8. Blue and red dots correspond to LS-PIV and SVD analysis, respectively. Analysis parameters were matched between LS-PIV and SVD using a 100 µm-long region of interest and the same processing time. Agreement between these methods was best at lower velocities and diverged with increasing speed. (TIF) [file pone.0038590.s004.tif]

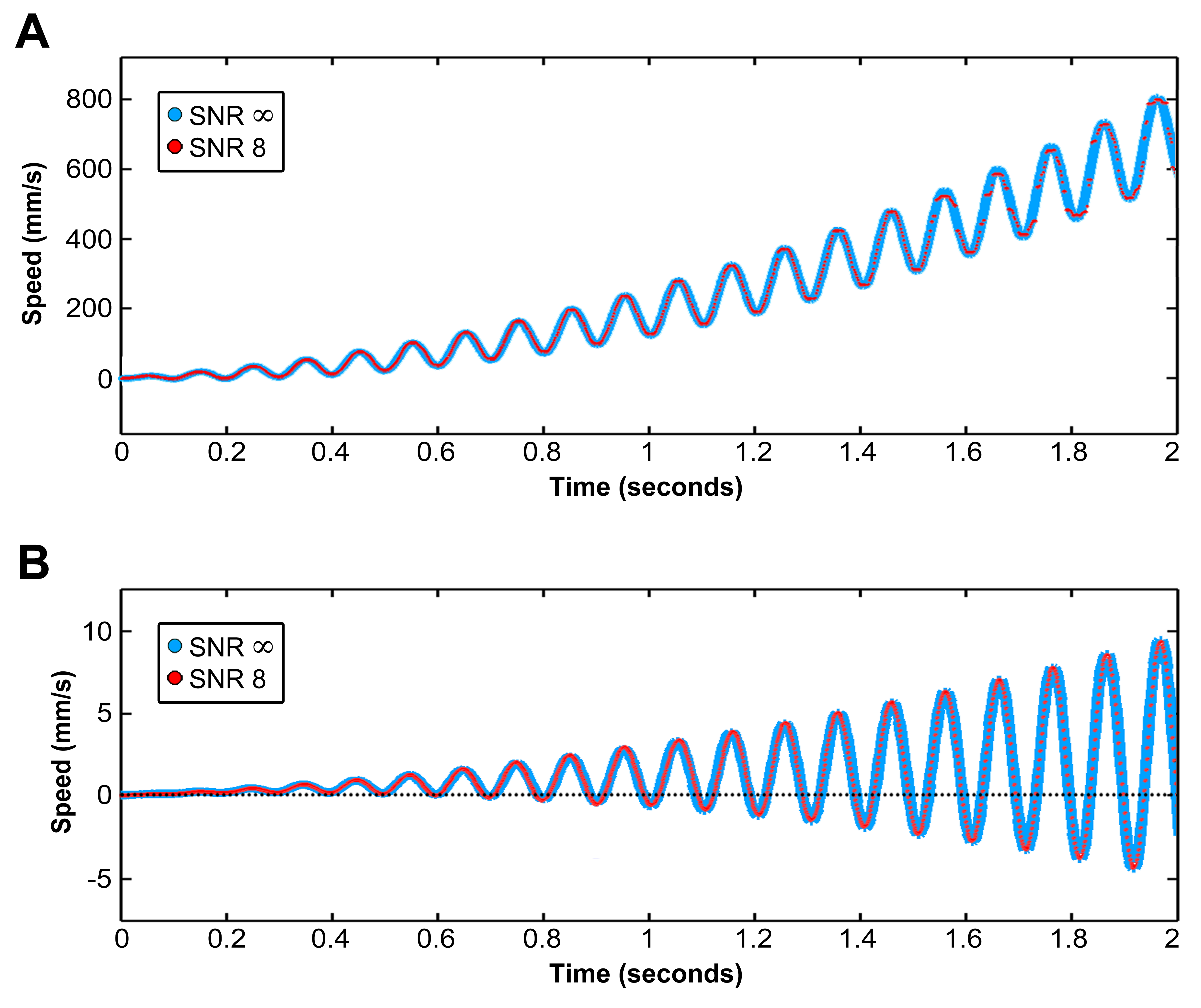

Supplement: Figure S5 — Performance of LS-PIV with high velocities and flow reversal. A, LS-PIV analysis of simulation line-scan data with velocity increasing to 800 mm/s. Actual particle velocity is represented by the blue line (SNR of infinity). Analysis was performed on data with an SNR of 8, within a 426 µm-long region of interest, and displayed as red dots. B, LS-PIV analysis on simulation line-scan data with increasing velocity and reversal of flow. Actual particle velocity is represented by the blue line (SNR of infinity). Analysis was performed on data with an SNR of 8 and displayed as red dots. (TIF) [file pone.0038590.s005.tif]

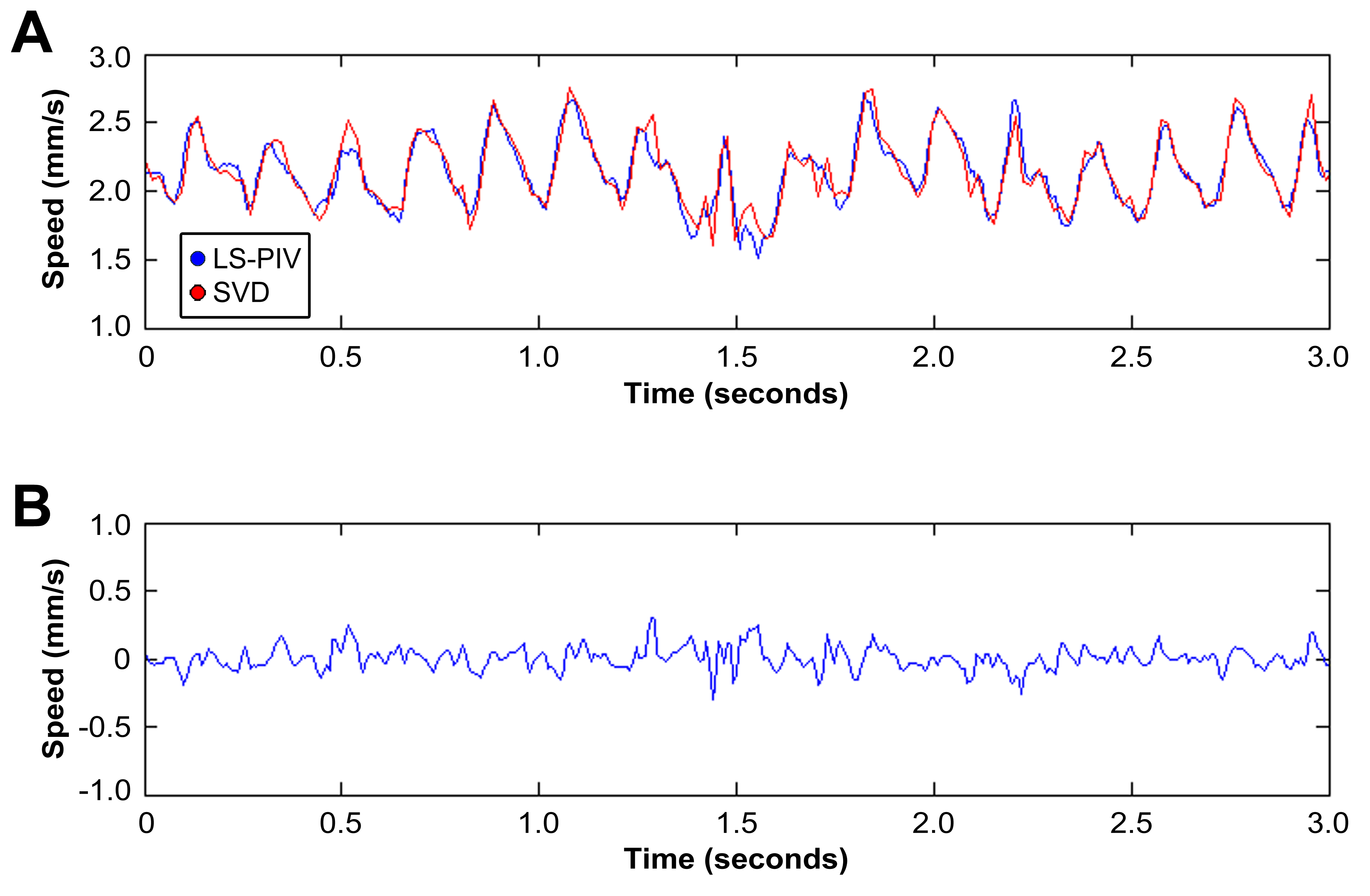

Supplement: Figure S6 — Analyses of in vivo capillary data by LS-PIV and SVD. A, Analyses of in vivo line-scan data using LS-PIV (blue line) demonstrating good agreement with SVD (red line), the gold standard, on capillary flow. In this example, cross-correlations for LS-PIV were conducted between every line-scan and its 5th neighboring scan. B, Plot of the difference in velocities calculated by SVD and LS-PIV analysis, corresponding in time with panel (A). The difference corresponds to 4.9% of the standard deviation of the average velocity determined by LS-PIV. (TIF) [file pone.0038590.s006.tif]

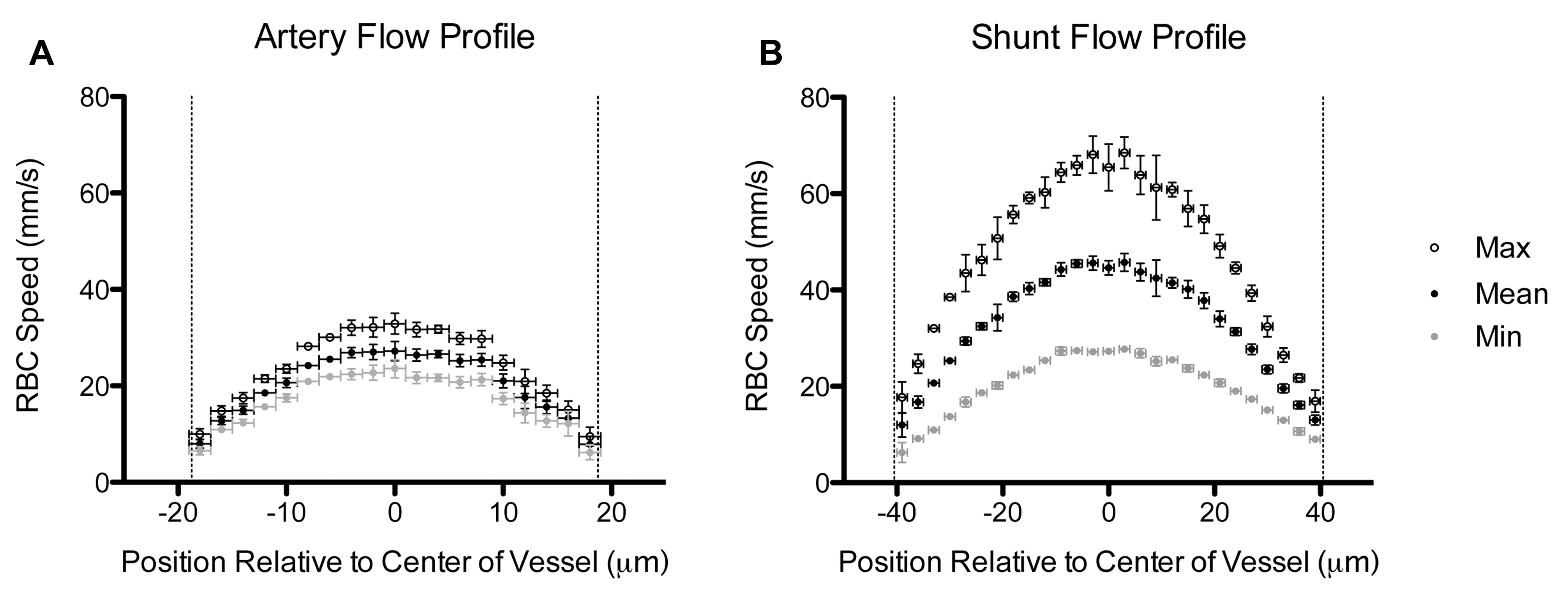

Supplement: Figure S7 — Cross-sectional flow profiles in control artery and AV shunt. A, RBC speed as a function of transverse position in a control artery. B, RBC speed as a function of transverse position in an AV shunt. Maximum, mean, and minimum RBC speeds were calculated from pulsatile velocities across 5 s scans. Five separate datasets were analyzed at each position in the vessel to determine the final velocities and their standard deviations (vertical error bars). Measurements were acquired serially along the vessel diameter over the span of ∼15 minutes. Vertical dotted lines represent the position of the vessel walls. Interestingly, measured RBC velocities did not go to zero at the wall, likely because of the cells’ finite size and inability to move closer to the boundary than half their width. Positional error was estimated additively from the optical resolution, translation stage jitter, and motion artifact of the animals (horizontal error bars). (TIF) [file pone.0038590.s007.tif]
