# Supplementary material for: Line-Scanning Particle Image Velocimetry: An Optical Approach for Quantifying a Wide Range of Blood Flow Speeds in Live Animals
Source: PLoS One. 2012 Jun 26;7(6):e38590. doi: 10.1371/journal.pone.0038590 (PMC3383695; doi:10.1371/journal.pone.0038590)
Supplement: Text S1 — Example Line-scan Data and LS-PIV Matlab Implementation. (PDF) [file pone.0038590.s008.pdf]

Supplemental Material

Example Line-scan Data and LS-PIV Matlab Implementation

Example data-sets for arterial and capillary flow have been included as supplemental files. Currently, the software imports multi-frame tiff files (a common format for many TPLSM systems). We convert this data into a single image matrix with continuous time along the rows and position along the columns. The software can be easily modified to import other file formats using standard Matlab library functions. Note that the spatial and temporal values of each pixel will depend on the experimental setup. In the example data provided, each pixel along the x and y axes correspond to ~0.277 μm and ~0.385 ms, respectively.

Two annotated versions of the LS-PIV Matlab code are provided as supplemental files. The 'LSPIV' script utilizes a single processor core for analysis and also provides a running depiction of the Gaussian fits to the cross-correlation peaks. The 'LSPIV_parallel' script allows multiple cores to enable faster analysis, where 'numWorkers' should be set to the number of cores on a particular system. Before running either script, three parameters should be selected to adjust the desired resolution and speed of analysis, and to accommodate slow or fast data-sets. 'Skipamt' inversely reduces the number of velocity points analyzed and increases the speed of analysis. 'Numavgs' averages the selected number of neighboring cross-correlations to increase SNR. Care should be taken not to average excessively, as this will artificially soften the peaks and troughs of pulsatile flow. 'Shiftamt' determines cross-correlation between Nth neighboring line-scans and should be increased for capillary flows where the displacement of RBCs is small relative to the pixel resolution of the line-scans. 'Shiftamt' should be set to 1 for fast flows.

The pulsatile velocities from *in vivo* data are an excellent indicator for successful analysis. Erroneous velocity calculations from cross-correlations tend to present with high or low values. Two additional parameters may be selected to flag these points in the Matlab code. 'Numstd' sets a threshold of standard deviations from the mean of analysis before flagging results. 'Windowsize' defines the number of scans in a window that is used to determine a running mean. Choose the velocity threshold and window size as needed for experiments. A simple application is to set the velocity threshold at ~3 standard deviations, and a time window that incorporates multiple cardiac cycles.

Upon running the matlab scripts, the graphical interface will request which settings to use, which specific data-set, and then prompt the user to select the region of interest within the corresponding space-time data. Good boundary pixel values along the x axis are [200, 300] for the 'capillary', [200, 300] for the 'artery', and [100, 400] for the 'fastartery' examples. Completed analysis will depict the raw data with time aligned along the x axis, a composite of the cross-correlations, and Gaussian-fitted displacements (Figure S3). Note that these results are reported in units of 'pixels' for both displacement and time. The displacements should be converted to units of distance and then divided by the time of one scan interval to convert to true velocity.

Example data-sets and annotated Matlab code are available at:
https://sourceforge.net/projects/lspivsupplement/files/
